# Bowel Rest with Total Parenteral Nutrition as an Alternative to Diverting Ileostomy in High-Risk Colorectal Anastomosis: A Pilot Study

**DOI:** 10.3390/medicina58040510

**Published:** 2022-04-02

**Authors:** Marius Kryzauskas, Matas Jakubauskas, Neda Gendvilaite, Vilius Rudaitis, Tomas Poskus

**Affiliations:** 1 Clinic of Gastroenterology, Nephrourology, and Surgery, Institute of Clinical Medicine, Faculty of Medicine, Vilnius University, LT-03101 Vilnius, Lithuania; marius.kryzauskas@santa.lt (M.K.); tomas.poskus@santa.lt (T.P.); 2 Faculty of Medicine, Vilnius University, LT-03101 Vilnius, Lithuania; neda.gendvilaite@mf.stud.vu.lt; 3 Clinic of Obstetrics and Gynecology, Institute of Clinical Medicine, Faculty of Medicine, Vilnius University, LT-08661 Vilnius, Lithuania; vilius.rudaitis@santa.lt

**Keywords:** anastomotic leakage, total parenteral nutrition, bowel rest, colorectal surgery

## Abstract

Anastomotic leakage remains the most feared complication in colorectal surgery. Various intraoperative tests evaluate bowel perfusion and mechanical integrity of the colorectal anastomosis. These tests reduce the risk of postoperative anastomotic leakage; however, the incidence remains high. Diverting loop ileostomy mitigates the damage if anastomotic leakage occurs. Nevertheless, ileostomy has a significant rate of complications, reducing patients’ quality of life, and requiring an additional operation. We evaluated six consecutive cases where bowel rest with total parenteral nutrition was used instead of diverting loop ileostomy. All colorectal anastomoses were at high risk of postoperative anastomotic leakage. Total parenteral nutrition was administered for the first seven days postoperatively. There were no serious complications during the recovery period, and no clinical postoperative anastomotic leakage was detected. All patients tolerated total parenteral nutrition. Bowel rest with total parenteral nutrition may be a feasible option in high-risk left-sided colorectal anastomosis and a possible alternative to a preventive loop ileostomy. Further studies are necessary to evaluate it on a larger scale.

## 1. Introduction

Anastomotic leakage (AL) remains one of the most feared complications in colorectal surgery. Despite immense research effort and practice changes, the percentage of AL remains high [1]. A diverting loop ileostomy is often used for damage control if AL occurs. However, ileostomies may cause complications, they significantly reduce patients’ quality of life and require an additional operation to close [2,3]. Moreover, up to 20 percent of preventive ileostomies are never closed [4]. Total parenteral nutrition (TPN) was previously adopted for patients with confirmed AL after upper gastrointestinal tract surgery [5]. However, it was never widely adopted in colorectal surgery and was only described as a one-off case in the literature [6]. Therefore, we hypothesised that short-term bowel rest with TPN could replace diverting loop ileostomy in high-risk left-sided colorectal anastomoses.

## 2. Materials and Methods

Consecutive patients undergoing elective left-sided colorectal resection with high-risk primary anastomosis (anastomosis ≤ 10 cm from the anal verge and/or presence of severe, life-threatening comorbidity) who agreed to participate were included. The central venous line was placed during anaesthesia, and patients underwent bowel rest with TPN for the first seven postoperative days.

TPN consisted of 1477 mL SmofKabiven, 10 mL of Addaven, 10 mL of Soluvit N, and 10 mL of Vitalipid N. The infusion starting speed was 30 mL/hour on the first postoperative day, 45 mL/hour on the second, and 62 mL/hour on the third until the seventh postoperative day.

Complete blood count, C-reactive protein (CRP), and electrolyte concentrations were monitored daily. After TPN, on the eighth postoperative day, patients were allowed to drink and eat liquid food.

The primary outcome of the study was the AL rate. Secondary outcomes included postoperative morbidity rate and tolerance of TPN. Postoperative complications were graded by the Clavien–Dindo classification.

## 3. Results

Six patients were included in the study. Patient details are described in Table 1. There was no clinical postoperative AL detected. Two patients had elevated CRP during the parenteral nutrition period. Chest, abdomen, and pelvic computed tomography scans with enteric contrast were performed, and AL was ruled out. These two patients developed grade II Clavien–Dindo complications: One patient developed postoperative fever, with negative blood and urine cultures, and the other developed wound seroma requiring drainage. Intravenous antibiotics were prescribed and inflammatory markers normalised. All other four patients had an uneventful postoperative course. All six patients did not have any complications associated with TPN.

## 4. Discussion

We present an alternative to diverting loop ileostomy by using bowel rest and TPN in high-risk left-sided colorectal anastomoses. There was no AL detected, and all patients tolerated bowel rest with TPN.

Colorectal surgeons aim to create safe anastomosis by ensuring adequate bowel perfusion and mechanical integrity of the anastomosis. Several studies showed the benefit of bowel perfusion (indocyanine green) and mechanical integrity (air-leak and methylene blue) testing in reducing postoperative AL [1,7,8]. Unfortunately, the risk of postoperative AL remains quite high even though anastomosis mechanical integrity and bowel perfusion are ensured. Thus, preventive ileostomy remains relevant to reducing the risk and consequences of AL [9,10]. Bowel rest and TPN achieve the same goal—dysfunction colorectal anastomosis—but it avoids repeated operation, necessary for an ileostomy.

One of the recommendations for enhanced recovery after surgery (ERAS) is early postoperative oral nutrition [11]. Therefore, bowel rest with TPN is contradictory to current ERAS guidelines. However, an ileostomy is a significant burden for the patient, reducing the quality of life, and is one of the most undesirable effects of colorectal surgery Furthermore, in most cases, a diverting ileostomy needs additional hospitalisation to revert it. This type of surgery has its own complications, some of them even being life-threatening [12]. Some surgeons are even arguing against the routine use of diverting ileostomy due to the high long-term morbidity associated with it [13]. Taking all this into account, patients eagerly agreed to participate in the study when avoiding ileostomy was an option.

## 5. Conclusions

In conclusion, bowel rest with total parenteral nutrition may be a feasible option in high-risk left-sided colorectal anastomosis and a possible alternative to a preventive loop ileostomy. Further studies are necessary to evaluate it on a larger scale.

## Figures and Tables

**Table 1:** Detailed patients, surgery, and outcomes characteristics.

Patient	Age	ASA	Gender (M/F)	BMI	Risk Factors	Indication for Surgery	Surgery (Open/Laparoscopic)	Indications for Ileostomy	Highest CRP (mg/L)	Postoperative Complications
1	55	II	F	25.6	Carcinoma of the fallopian tube	Carcinoma penetrating the rectal wall	Open	Low anastomosis (8 cm from anal verge) Positive methylene blue test	56.6	None
2	55	III	F	43.5	Morbid obesity	Carcinoma of the sigmoid colon	Laparoscopic converted to open	Low anastomosis (10 cm from anal verge) Obesity	219.8	Postoperative wound seroma
3	61	III	F	23.1	Acute renal failure Hypokalaemia Hyponatraemia Sepsis	Adenoma of the sigmoid colon (McKittrick–Wheelock syndrome)	Laparoscopic	Renal failure	181.9	Postoperative fever (second postoperative day)
4	77	III	F	33.2	Disseminated carcinoma of the uterus	Uterine carcinoma penetrating the rectal wall	Open	Low anastomosis (5 cm from anal verge)	71.7	None
5	50	IIIE	M	40.9	Chronic renal failure Haemodialysis Morbid obesity	Rectal carcinoma	Laparoscopic	Low anastomosis (7 cm from anal verge) Obesity Renal failure	43.3	None
6	43	II	M	22.6	Neoadjuvant chemoradiation	Rectal carcinoma	Laparoscopic	Low anastomosis (2 cm from anal verge)	21.3	None

ASA: American Society of Anesthesiology score; M/F: Male/Female; BMI: Body mass index; CRP: C-reactive protein.

## Data Availability

Data are available on reasonable request. All data relevant to the study are included in the article. Deidentified data that underlie the results reported in this article will be shared with third parties after a written request to the corresponding author describing the intention of data usage and full affiliation of the requesting organisation. To gain access to the data, a data access agreement needs to be signed.
